# Hemophagocytic Lymphohistiocytosis in the Setting of Disseminated Histoplasmosis and Uncontrolled HIV

**DOI:** 10.7759/cureus.24360

**Published:** 2022-04-21

**Authors:** Brandon C Warren, Harika Yadav, Matthew Graham, Maria Tudor

**Affiliations:** 1 Internal Medicine, University of Tennessee, Chattanooga, USA; 2 Hematology/Oncology, Tennessee Oncology, Chattanooga, USA; 3 Internal Medicine, The University of Tennessee Health Science Center at Chattanooga, Chattanooga, USA

**Keywords:** macrophage activation syndrome (mas), uncontrolled hiv, histoplasmosis, hiv-positive, hemophagocytic lymphohistiocytosis (hlh)

## Abstract

Hemophagocytic lymphohistiocytosis (HLH) is a rare and life-threateningly aggressive syndrome caused by excessive immune activation. It involves the abnormal activation of lymphocytes and macrophages which leads to tissue destruction and inflammation. Traditionally HLH classification is currently separated into primary and secondary HLH based on genetic versus nongenetic events such as infection, malignancy, or autoimmune disorders. In this case report, we present the case of a middle-aged woman presenting with HIV with medication noncompliance who presented to the emergency department with pancytopenia as well as disseminated histoplasmosis and was diagnosed with HLH based on the HLH-2004 guidelines and treated in accordance with the HLH-94 protocol. The patient also underwent treatment for the management of her histoplasmosis with a favorable outcome. This case demonstrates that HLH is best treated through management of the underlying process that triggered the syndrome such as infection as in this patient in addition to management per HLH-94 protocol early on in the course of the disease in order to have the best chance at a positive clinical outcome.

## Introduction

In the setting of disseminated histoplasmosis secondary to an uncontrolled human immunodeficiency virus (HIV) infection, many patients will be febrile with an elevated ferritin level and lactate dehydrogenase (LDH). A bi- or pancytopenia should raise concern for hemophagocytic lymphohistiocytosis (HLH), a rare condition that has two variants, a primary HLH type found in pediatric patients generally due to genetic mutations and a secondary type found in malignancy, infection and autoimmune disorders. Newer classification guidelines per the North American Consortium for Histiocytosis (NACHO) however state that these classifications should not be used as primary HLH can be triggered by immune-activating events or infection, while genetic mutations can be found in individuals at any age [1]. For the purposes of this article, we will continue to use the classical classifications of primary and secondary HLH. Here we present a 42-year-old female patient with a history of uncontrolled HIV found to have disseminated histoplasmosis on admission to the hospital found to have a pancytopenia with an elevated ferritin level and D-Dimer, concerning for secondary HLH based on the HLH-2004 guidelines [2].

## Case presentation

We present a 42-year-old femalewith a past medical history of HIV with medication noncompliance, treated active tuberculosis in 2006, and depression who initially presented to the emergency department with a chief complaint of urinary incontinence, who was found to have pancytopenia. A review of systems was positive for fatigue, shortness of breath, and loss of appetite for the last 2 months. Of note, she also had two new sexual partners over the last year. On further questioning, the patient revealed that she had a history of polysubstance abuse including intravenous drug use and recent active genital herpes undergoing treatment with acyclovir. 

**Figure 1:**
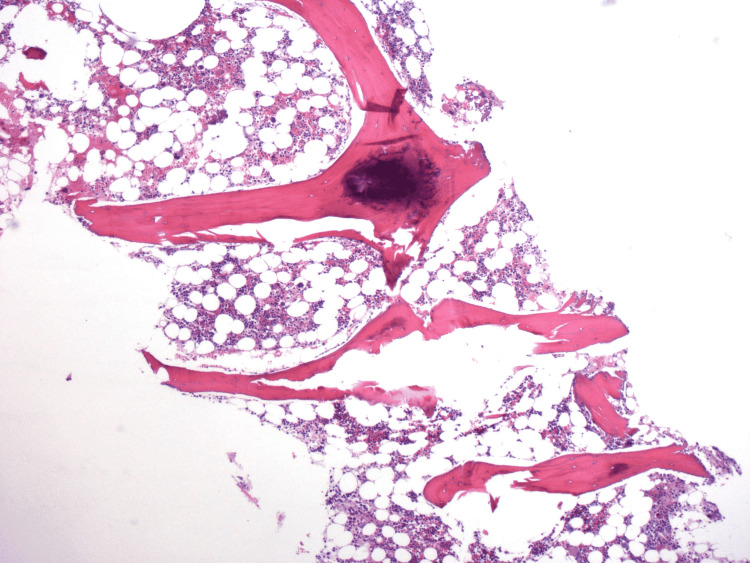
Bone marrow biopsy showing hypocellular marrow for age and abundant histiocytes with intracellular organisms.

On admission, she had pancytopenia with a hemoglobin of 6.4 g/dL, a White blood cell count of 0.7 k/mm³, and a platelet count of 8k/mm³ leading to a subsequent admission to the intensive care unit. She underwent a peripheral smear and bone marrow biopsy which showed findings of lymphohistiocytic infiltrates and inclusions (Figure 1). With concern for possible HLH, the patient’s fibrinogen level was checked and was noted to be low at 89 mg/dL. In addition, she had an elevated D-dimer of 16370 ng/mL and an elevated ferritin of more than 15,000 ng/mL. She was also noted to have splenomegaly on physical exam. Finally, the patient was also noted to have a positive urine enzyme immunoassay test for histoplasmosis. Her pancytopenia was concerning for macrophage activation syndrome (MAS) and hematology was consulted for evaluation. This was later confirmed by hemophagocytosis noted on bone marrow biopsy. Due to concern for worsening sepsis, the patient was immediately started on amphotericin B for management of her histoplasmosis.

She subsequently received multiple red blood cell (RBC) and platelet transfusions, without much improvement in her cell counts over the initial three-day period after admission. She was then started on high-dose dexamethasone with taper and was found to have rapid improvement in her cell counts. Because of her untreated HIV, she was started on bactrim for antibiotic prophylaxis and initiation of highly active antiretroviral therapy (HAART) while inpatient in addition to valacyclovir for the treatment of genital HSV. The patient’s cell lines including her white blood cells (WBC), RBC, and platelets all returned to normal baseline values and her clinical symptoms rapidly improved to her baseline. After 11 days from her initial admission, she was deemed stable for discharge from the hospital with a follow-up scheduled with her hematologist.

## Discussion

Hemophagocytic lymphohistiocytosis (HLH) is a disease of abnormal immune activation. It involves the abnormal activation of lymphocytes and macrophages with overproduction of cytokines [3-4]. Because these natural killer cells as well as cytotoxic t-lymphocytes fail to eliminate activated macrophages, excessive release of cytokines can result, and have the potential to develop a cytokine storm. This can lead to multiorgan system failure and potentially death [3].

Classification of HLH is normally separated into primary and secondary HLH. Primary HLH was thought to result from an autosomal recessive disorder with multiple subtypes based on the genetic abnormality associated with the disorder. This was thought to be the most common form of HLH and is responsible for the form of the disease most commonly found in children. In contrast, secondary HLH was associated with an acquired form of the disease based on conditions such as malignancy, infection, or other autoimmune disorders [5-7]. Newer guidelines per NACHO however state that this classification system should no longer be used as there is an overlap between primary and secondary HLH as the interaction between genetic mutations and infectious, malignant, or autoimmune disorders may overlap.

Diagnosis of HLH is based on the HLH-2004 criteria and includes at least five of the criteria listed in Table 1 [2].

**Table 1:** HLH-2004 guidelines on criteria for the diagnosis of HLH HLH: Hemophagocytic lymphohistiocytosis; NK cell: natural killer cell; IL: interleukin

HLH-2004 laboratory or clinical criteria	Value
Fever	peak temperature of > 38.5° C for > 7 days
Splenomegaly	spleen palpable > 3 cm below costal margin
Cytopenia	Involving > two cell lines (hemoglobin < 9 g/dL [90 g/L], absolute neutrophil count < 100/mcL [0.10 × 10^9^/L], platelets < 100,000/mcL [100 × 10^9^/L])
Hypertriglyceridemia or hypofibrinogenemia	Fasting triglycerides > 177 mg/dL [2.0 mmol/L] or > 3 standard deviations (SD) more than normal value for age or fibrinogen 3 SD less than normal value for age
Hemophagocytosis	Noted on peripheral smear or bone marrow, spleen, or lymph node biopsy
NK cell activity	Low or absent
Serum ferritin	>500 ng/mL
Elevated IL-2 (CD25) level	(CD25) levels (>2400 U/mL or very high for age)

This case demonstrates the rare occurrence of HLH in the setting of a patient with uncontrolled HIV found to have disseminated histoplasmosis. Because disseminated histoplasmosis also may present with fevers and elevated ferritin levels with a similar presentation to HLH, some providers may not immediately decide to undergo further testing for further workup for HLH which would normally include soluble interleukin-2 receptor alpha (IL-2Rα), interleukin-18 (IL-18), and chemokine ligand 9 (CCL-9). In addition, patients with concern for HLH should have a bone marrow aspiration performed to evaluate for the cause of cytopenia and to detect hemophagocytosis, which is reported in 25-100% of cases of HLH [8].

The primary treatment for secondary HLH is the management of the patient’s inciting condition, such as disseminated histoplasmosis in this patient. In the secondary form of HLH, immunosuppression is often required for clinical improvement. This should be applied in a stepwise fashion. Dexamethasone is typically the front-line immunosuppressant utilized in these cases per the HLH-94 treatment protocol [9]. Before adding additional therapies, a full trial of dexamethasone should be utilized. If unsuccessful, etoposide and cyclosporine can be carefully considered.

Inflammatory syndrome (IRIS) is a disorder in which patients experience paradoxical worsening of infectious symptoms with the restoration of the immune system following initiation of HAART in patients with uncontrolled HIV [10]. In patients taking HAART for uncontrolled HIV with HLH, the clinician should consider waiting to initiate HAART until the patient’s acute phase of HLH has resolved.

## Conclusions

In patients presenting with immunosuppression from uncontrolled HIV, any bi- or pancytopenia should immediately raise concern to include HLH in the differential diagnosis. Opportunistic infections such as histoplasmosis as found in this patient have been noted in literature reviews to contribute to inappropriate immune activation. Clinical management early in the course of the disease with the management of the inciting infection or any other event in addition to treatment per the HLH-94 treatment protocol will give the patient the best chance of recovery in a disease that has a documented adult mortality rate of 41-75% if left untreated.
